# The effect of early versus delayed surgical debridement on the outcome of open long bone fractures at Bugando Medical Centre, Mwanza, Tanzania

**DOI:** 10.1186/s13032-016-0036-7

**Published:** 2016-07-04

**Authors:** Njee Nobert, Nyambura Moremi, Jeremiah Seni, Ramesh M. Dass, Isdori H. Ngayomela, Stephen E. Mshana, Japhet M. Gilyoma

**Affiliations:** 1 Department of Surgery, Catholic University of Health and Allied Sciences, P.O.Box 1464, Mwanza, Tanzania; 2 Department of Surgery, Bugando Medical Centre, P.O.Box 1370, Mwanza, Tanzania; 3 Department of Microbiology and Immunology, Catholic University of Health and Allied Sciences, P.O.Box 1464, Mwanza, Tanzania

**Keywords:** Open fractures, Surgical debridement, Outcomes, Mwanza

## Abstract

**Background:**

Urgent surgical debridement of open long bone fractures is of paramount importance for prevention of subsequent infection. Due to limited information on the timing of this surgical procedure in Mwanza, Tanzania; the present study was conducted to evaluate the effect of early versus delayed surgical debridement on the outcome of open long bone fractures.

**Methods:**

A prospective cohort study involving 143 patients with open long bone fractures admitted at Bugando Medical Centre (BMC) between December 2014 and April 2015 was conducted. Patients were stratified into two main groups basing on whether they presented at BMC and operated early (within 6 h) or late (more than 6 h). Socio-demographic and clinical information were collected using structured questionnaire. Analysis was done using STATA software version 11.

**Results:**

The male to female ratio was 1.6: 1, with most of the patients being in their third decade of life (30.8 %). Road traffic accident (RTA) was the most common cause of fractures (67.8 %). Majority of patients, 91 (63.6 %) had Gustillo-Anderson grade II and the timing of debridement was significantly associated with this grading (*p*-value = 0.05). Nine (6.3 %) patients developed surgical site infection (SSI) and the median length of hospital stay (LOS) (interquartile range) was 7 (5–10) days, ranging from 3 to 35 days. SSI was found more in the late group compared to the early group [7.5 % (6/80) versus 4.8 % (3/63) respectively, *p*-value = 0.503)] and LOS was also longer in the late group compared to the early group [7 (6–11.5) days and 6 (5–10) days respectively, *p*-value = 0.06]. *Pseudomonas aeruginosa* was the predominant bacteria causing SSI.

**Conclusions:**

Open long bone fracture injuries due to RTA are common at BMC. The risk of developing SSI in this setting is low and comparable to many other countries. Despite the fact that there was no statistical significant difference between early versus delayed debrided groups on SSI and LOS stays; the need for prompt surgical intervention in both groups should be an enduring focus to maintain these favorable outcomes.

## Background

The growing burden of open fractures is apparently of global concern and contribute significantly to high morbidity and long-term disabilities [1–4]. It is estimated that the annual incidence of open fractures is 11.5 per 100 000 persons with preponderance of lower extremities [1]. In Tanzania and other countries in Sub Saharan Africa, the burden of open fractures has a long historical background but is currently escalating due to legalization of motor cycles as a means of public transport [2, 5–7]. Moreover, the negative health impact related to open fractures due to road traffic accidents (RTA) are many ranging from increased hospital admissions in the intensive care unit, infections, longer duration of hospital stays, long term disabilities and deaths [2, 8, 9].

In the light of the increase in the trends of open fractures in Tanzania, there is an obvious need to revisit the existing management guidelines so as to ensure better outcomes among patients involved. Despite the fact that there are advances in technological innovations on the management of open fractures compared to conventional management modalities, there are still numerous challenges especially in developing countries [10–13]. The long debated optimal timing of surgical debridement basing on the “6 h rule” still remain to be controversy as some studies support it whereas others refute it to be associated with plausible outcome in terms of less incidences of surgical site infections and shorter LOS [13–20].

Therefore, the present study intended to establish evidence based and locally generated information on the effect of timing of surgical debridement as stipulated in the “6 h rule” on the outcome of patients with long open bone fractures at Bugando Medical Centre (BMC).

## Methods

### Study area, design and sampling procedures

This was a prospective cohort study conducted between December 2014 and April 2015 at accident & emergency department and orthopedic & traumatology wards at BMC which is a tertiary, consultant and teaching hospital serving over 13 million people in the Lake Victoria region - North-western Tanzania. The study involved all patients with open long bone fractures admitted at BMC who voluntarily consented to participate in the study and subsequently underwent surgical debridement in the operating theatres. The study excluded patients with open long bone fractures and other life-threatening injuries such as head, chest, or abdominal injuries as these would be the priority groups in terms of management over any limb-threatening injury as well as patients with overt clinical signs of infections. The sample size of 148 patients was calculated using Kirkwood and Sterne (2003) at 5 % significance level, power of 90 % and based on the proportion of 11.6 % from a previous study [18]. Convenience sampling of patients who meet the inclusion criteria was performed until the sample size was reached.

### Patients’ management and data collection

Patients were informed about the study and requested to voluntarily participate. A structured questionnaire was administered which in turn stratified them into two main groups basing on whether they presented at BMC and operated early (within 6 h) or late (more than 6 h). All patients were managed according to BMC management protocol on open long bone fractures which included Advanced Trauma Life Support, urgent baseline investigations (Hemoglobin estimation, blood grouping and cross matching); radiological tests (x-rays of the affected bone); provision of systemic antibiotic prophylaxis (Ceftriaxone and Metronidazole) and anti tetanus prophylaxis; grading of open long fracture according to Gustilo-Anderson classification system [10]; surgical debridement in the theatre followed by fracture stabilization by Plaster of Paris cast (POP), external fixation and open reduction and internal fixation (ORIF), and early soft-tissue coverage whenever required.

In the ward, the provider initiated counselling and testing for HIV serological tests was performed according to the National Guideline [21]. Patients were followed up to assess the SSI incidences. SSI was defined as previously described as the presence infection which occurs within 30 days after the operation evidenced by purulent discharge from the site, isolation of bacterial pathogen(s) and at least one of the following signs or symptoms of infection namely pain or tenderness, localized swelling, redness, or heat [22]. For patients who developed SSI, a swab with Stuart’s Transport media (Improswab®, Shanghai, China) was used to collect pus specimen basing on the standard guidelines and transported to the Catholic University of Health and Allied Sciences (CUHAS) Multipurpose Laboratory within an hour of collection to be analyzed. In the laboratory, the specimens were registered in the log book and processed based on standard microbiological procedures namely Gram stain, culture on Blood agar and MacConkey agar (HI Media, India) followed by biochemical identification tests according to the previously described methods [23]. The bacterial isolates were subjected to the antimicrobial susceptibility testing according to the Clinical Laboratory Standard Institute [24]. Patients were followed for 30 days to assess the incidence of SSI weekly during wound dressing and LOS in the two groups of patients [22].

### Data collection and analysis

Sociodemographic, clinical and laboratory data were collected using a structured questionnaire, transferred to Excel for consistence checks and analyzed using STATA version 11 according to the objectives of the study. Continuous variables were summarized using proportions and frequencies; and depending on the distribution of data, mean ± standard deviation or median (interquartile range) for continuous variable were used. Chi-square or Fischer’s exact tests was used to assess the distribution of data and p-value of less than 0.05 was used as a cuff off point for showing the significant association between predictor variables and outcome among patients with open long bone fractures. The main primary outcomes were occurrence of SSI and LOS.

### Ethical considerations

The approval to carry out this study was sought from the joint CUHAS/BMC Research, Ethics and publication committee. Voluntary written informed consent was sought from every participants prior to be involved into the study. Patient’s refusal to consent or withdraw from the study did not alter or jeopardize their access to standard medical care at BMC. Confidentiality was ensured by using anonymous codes. For patients who developed SSI, results for antimicrobial susceptibility testing was promptly given to the attending doctor for specific management.

## Results

**Fig. 1:**
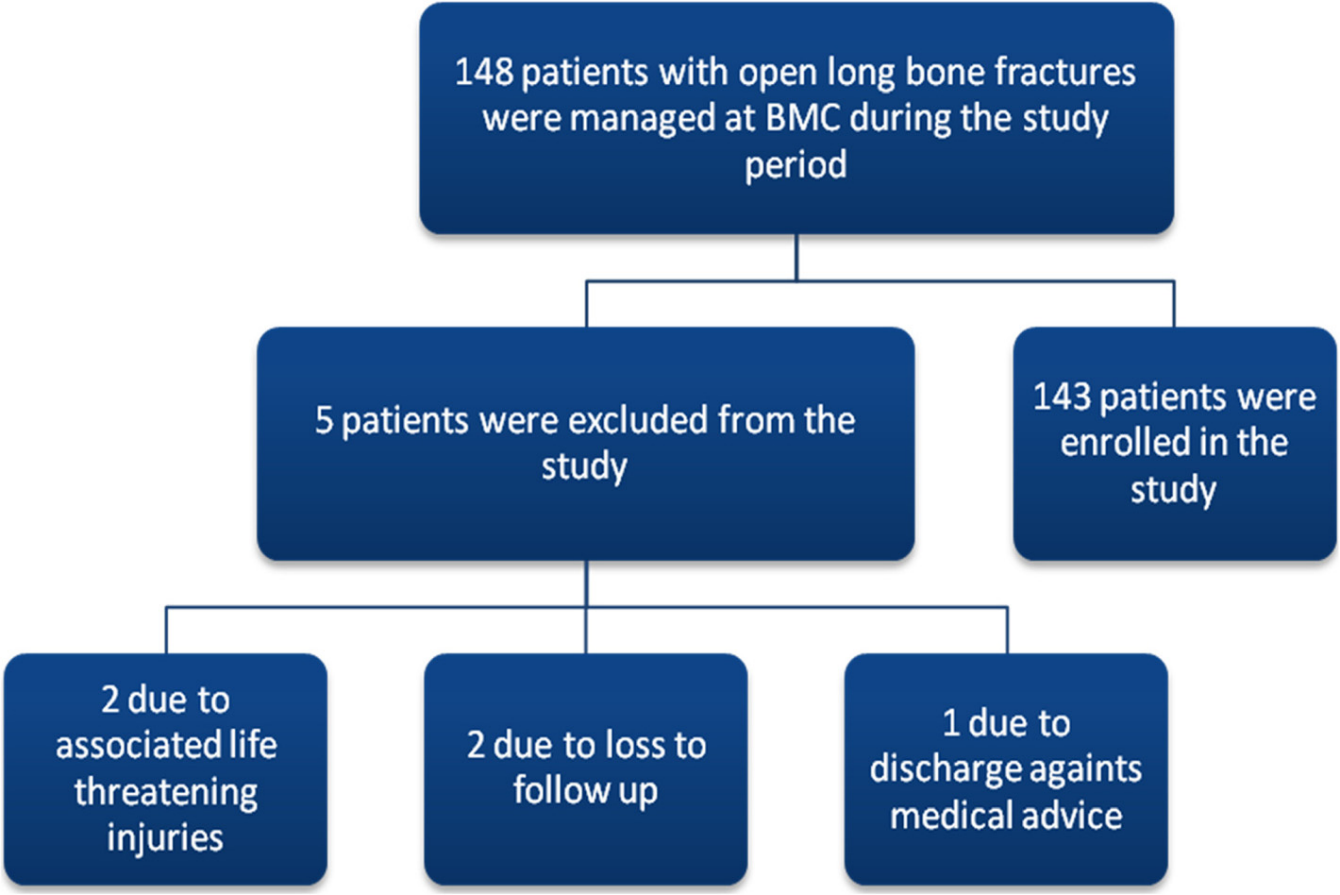
Flow chart showing enrollment process of study participants. BMC = Bugando Medical Centre

During the study period, a total number of 148 patients with open long bone fractures were managed at BMC. Of these, 5 patients were excluded from the study due to failure to meet the inclusion criteria (Fig. 1).

### Baseline socio-demographic and clinical characteristics of patients

The median age (IQR) of patients was 34 years (25–42 years), ranging from 11 to 87 years with the peak age between 31 and 40 years in approximately 30.8 % of participants. Majority of participants were males, 89 (62.2 %); with primary and secondary school education, 114 (79.7 %); peasants, 48 (33.5 %); and their open fractures were due to RTA 96 (67.1 %). Out of the143 patients, nine (6.3 %) were found to be HIV seropositive (Table 1).

**Table 1:** Baseline socio-demographic and clinical characteristics of patients with open long bone fractures

Socio-demographic and clinical characteristics	Number	Percentage
Sex		
Male	89	62.2
Female	54	37.8
Education		
Tertiary education	18	12.6
Primary or secondary education	114	79.7
No formal education	11	7.7
Occupation		
Students	18	12.6
Peasants	48	33.6
Driver	3	2.1
Motorcycle rider	19	13.3
Civil servants	19	13.3
Businessman/woman	34	23.8
Others	2	1.4
Cause of injury		
Road traffic accident	97	67.8
Falls	22	15.4
Assaults	20	14.0
Others	4	2.8
Place of injury		
Along the road	96	67.1
Residential areas (outside the house)	23	16.1
Home (within the house)	18	12.6
School	3	2.1
Other places	3	2.1
HIV serostatus		
Seropositive	9	6.3
Seronegative	134	93.7

### Distribution of fracture characteristics among patients with open long bone fracture

The tibia was the most common site of open fracture affecting 46.1 % of patients and majority, 91 (63.6 %) had Gustilo-Anderson grade II. The distal third and mid-shaft were the most frequent location of fracture accounting for 40.6 and 39.9 % of patients respectively and the most common fracture pattern was transverse in about 39.9 % (Table 2).

**Table 2:** Distribution of patients according to fracture characteristics

Fracture characteristics	Frequency	Percentages
Site of fracture		
Femur	31	21.7
Tibia	66	46.1
Ankle	5	3.5
Humerus	17	11.9
Forearm	19	13.3
Multiple	5	3.5
Gustilo Anderson grade		
I	24	16.8
II	92	63.3
IIIA	21	14.7
IIIB	5	3.5
IIIC	1	0.7
Location of fracture		
Proximal	21	14.7
Mid-shaft	57	39.9
Distal	58	40.6
More than one location	7	4.9
Affected limb		
Right	86	60.1
Left	55	38.5
Bilateral	1	1.4
Fracture pattern		
Transverse	57	39.9
Oblique	46	32.1
Comminuted	22	15.3
Spiral	18	12.6

### Treatment characteristics

Regarding timing of surgical debridement, 80 (55.9 %) patients had surgical debridement done 6 h post injury. The majority of surgical debridement, 138 (96.5 %) were performed by residents/medical officers and 5 (3.5 %) surgical debridement were performed by specialists.

Plaster of Paris (POP) was the most common method of skeletal stabilization performed in 70 (49.0 %) patients. This was followed by external fixator, skeletal traction and ORIF in 35 (24.5 %), 28 (19.6 %) and 9 (6.3 %) patients respectively. One (0.3 %) patient underwent limb amputation.

### Association of early versus delayed debridement with variables

There was no statistical association between socio-demographic and clinical characteristics of patients with the timing of surgical debridement. However, there was a significant association between Gustilo –Anderson grade and the timing of surgical debridement with majority of patients falling in Grade II [92 (63.3 %)] and of these 52.4 % (33/63) and 73.8 % (59/80) were debrided early and late respectively (*p*-value = 0.05) (Table 3).

**Table 3:** Association of early versus delayed debridement with variables

Variable	Surgical debridement		Chi 2	*p* value
	*N* = 143			
	Early (63)	Late (80)		
	n (%)	n (%)		
Median age^a^	32 (23–41)	35 (27–42)	-	0.4006
Sex				
Female	23 (31.5)	31 (38.8)	0.0754	0.784
Male	40 (63.5)	49 (61.2)		
Occupation				
Students	10 (15.8)	8 (10.0)	-	0.382
Peasants	20 (31.7)	28 (35.0)		
Driver	0 (0.0)	3 (3.7)		
Motorcycle rider	7 (11.1)	12 (15.0)		
Civil servants	10 (15.9)	9 (11.3)		
Businessman/women	14 (22.2)	20 (25)		
Others	2 (3.2)	0 (0.0)		
Cause of injury				
RTA	45 (71.4)	52 (65.0)	-	0.828
Falls	9 (14.3)	13 (16.3)		
Assaults	8 (12.7)	12 (15.0)		
Others	1 (1.6)	3 (3.75)		
Place of injury				
Along road	44 (69.8)	52 (65.0)	-	0.793
Residential area	10 (15.9)	13 (16.3)		
Home	6 (9.5)	12 (15.0)		
School	2 (3.2)	1 (1.2)		
Other places	1 (1.6)	2 (2.5)		
HIV Serostatus				
Seropositive	60 (95.2)	74 (92.5)	-	0.379
Seronegative	3 (4.7)	6 (7.5)		
Mechanism of injury				
Blunt injury	35 (55.6)	38 (47.5)	3.187	0.203
Penetrating	13 (20.6)	12 (15)		
Both	15 (23.8)	30 (37.5)		
Site of open fracture				
Femur	16 (25.4)	15 (18.8)	-	0.136
Tibia	27 (42.9)	39 (48.8)		
Ankle	0 (0.0)	5 (6.25)		
Humerus	9 (14.3)	8 (10.0)		
Forearm	7 (11.1)	12 (15.0)		
Multiple sites	4 (6.4)	1 (1.3)		
Gustillo and Anderson grade				
I	18 (28.6)	6 (7.5)	-	0.05
II	33 (52.4)	59 (73.8)		
IIIa	9 (14.3)	12 (15.0)		
IIIb	2 (3.2)	3 (3.8)		
IIIc	1 (1.6)	0 (0.0)		
Fracture location				
Proximal	10 (15.9)	11 (13.8)	-	0.865
Mid shaft	26 (41.2)	31 (38.8)		
Distal	25 (39.7)	33 (41.3)		
More than one location	2 (3.2)	5 (6.3)		
Fracture pattern				
Transverse	28 (44.4)	29 (36.3)	-	0.333
Comminuted	6 (9.5)	16 (20.0)		
Oblique	20 (31.8)	26 (32.5)		
Spiral	9 (14.3)	9 (11.3)		
Rank of surgeon				
Specialist	4 (6.4)	1 (1.3)	-	0.118
Resident/medical officer	59 (93.7)	79 (98.5)		
Skeletal stabilisation				
Skeletal traction	13 (20.6)	15 (18.8)	-	0.858
ORIF	5 (7.9)	4 (5.0)		
POP	29 (46.0)	41 (51.3)		
External fixation	16 (25.4)	20 (25.0)		

^a^Continuous variables

### Treatment outcomes

#### Surgical site infection

Out of 143 patients who underwent surgical debridement for open long bone fractures, nine (6.3 %) developed SSI. There was more SSI among patients who were debrided late compared to those debrided early (6/9 versus 3/9); *p*-value = 0.503, 95 % CI 1.62 (0.33–10.40) although the different was not statistically significant (Table 4).

**Table 4:** Association between the timing of surgical debridement and the rate of surgical site infections

Timing of surgical debridement	Surgical site infections		
	Present (n, %)	Absent (n, %)	Total (n, %)
Early (≤ 6 h of injury)	3 (33.3)	60 (44.8)	63 (44.1)
Late (> 6 h of injury)	6 (66.7)	74 (55.2)	80 (55.9)
Total	9 (100.0)	134 (100.0)	143 (100)

Pearson’s chi square (*X*2) = 0.448, *p*-value = 0.503

Majority of patients with SSI were in Gustilo grade II (4, 44.4 %), whereas Grade IIIA and IIIB had 3 (33.3 %) and 2 (22.2 %) patients respectively. The mechanism of injury among patients with SSI were blunt injury in about 6 (66.67 %) and a combination of both blunt and penetrating injuries in 3 (33.3 %). Most patients with SSI had open fracture involving the tibia (8, 88.9 %) whereas in one patient (11.1 %) humerus was involved.

### Length of hospital stay

The overall median length of hospital stay (IQR) was 7 (5–10) days with the range from 3 to 35 days. The median LOS (IQR) of patients who had surgical debridement within 6 h and after 6 h of injury were 6 (5–10) days and 7 (6–11.5) days respectively, *p*-value = 0.06. The respective ranges were 3 to 35 days and 3 to 23 days.

### The bacterial isolates involved in surgical site infections and their antimicrobial susceptibility patterns

Nine patients developed SSI resulting into 15 bacterial isolates on aerobic culture (five patients had single bacteria, two patients had two bacteria and two patients had three bacteria each). The most common bacteria was *Pseudomonas aeruginosa* 6 (40.0 %), followed by *Escherichia coli* 3 (20.0 %), *Klebsiella pneumonia* 3 (20.0 %), whereas *Proteus mirabilis*, *Pantoea agglomerans* and *Staphylococcus aureus* each constituted one isolate (6.6 %) (Table 5).

**Table 5:** Bacteria isolates causing surgical site infections in patients with open fracture and their antimicrobial susceptibility patterns

Gram negative bacteria:											
Bacterial isolates				Antimicrobial susceptibility patterns							
	Study #	AMP	SXT	GEN	CIP	FEP	TZP	CAZ	CTR	AM/C	MER
*Pseudomonas aeruginosa*	032	NA	NA	R	R	S	S	S	NA	NA	S
*Escherichia coli*	037	R	I	S	S	S	I	S	R	R	NT
*Klebsiella pneumoniae*	037	R	R	S	S	S	S	S	R	R	NT
*Proteus mirabilis*	037	R	S	S	S	S	NT	S	S	R	NT
*Pseudomonas aeruginosa*	095	NA	NA	S	S	NT	S	S	NA	NA	S
*Pseudomonas aeruginosa*	096	NA	NA	S	S	NT	S	I	NA	NA	S
*Pseudomonas aeruginosa*	099	NA	NA	S	S	NT	S	S	NA	NA	S
*Escherichia coli*	103	R	R	S	S	S	NT	S	S	R	S
*Klebsiella pneumoniae*	103	R	R	S	S	S	NT	S	S	S	S
*Pseudomonas aeruginosa*	110	NA	NA	S	S	NT	S	R	NA	NA	S
*Pseudomonas aeruginosa*	115	NA	NA	S	S	NT	S	S	NA	NA	S
*Klebsiella pneumoniae*	141	R	R	R	S	S	R	R	R	R	S
*Escherichia coli*	141	R	R	S	R	S	R	R	R	R	R
*Pantoea agglomerans*	141	R	R	S	S	NT	R	R	R	R	S
Gram positive bacteria:											
Bacterial Isolate	#	FOX	SXT	GEN	CIP	ERY	CAD	VAN			
*Staphylococccus aureus*	115	S	R	S	S	S	S	S			

*AMP* ampicillin, *SXT* trimethoprim-sulphamethoxazole, *GEN* gentamicin, *CIP* ciprofloxacin, *FEP* cefepime, *TZP* piperacillin-tazobactam, *CAZ* ceftazidime, *CTR* ceftriaxone, *AMC* amoxycillin-clavulanate, *MER* meropenem, *FOX* cefoxitin, *ERY* erythromycin, *CAD* clindamycin, *VAN* vancomycin, *S* sensitive; *R* resistance, *I* intermidiate, *NA* not applicable, *NT* not tested

There was high level of resistance among tested Gram negative bacteria to ampicillin (100 %, 8/8), trimethoprim sulphamethoxazole (87.3 %, 7/8) and ceftriaxone (62.5 %, 5/8). Low resistance to gentamicin (14.3 %, 2/14), ciprofloxacin (14.3 %, 2/14), cefepime (0 %) and meropenem (9.1 %, 1/11). *Staphylococcus aureus* isolate was sensitive to all antimicrobial agents tested except for Trimethoprim sulphamethoxazole (Table 5).

## Discussion

In this study, most of patients were youth in their most productive years and showed a male preponderance. Similar demographic observations were also reported by other authors [2, 6, 7, 25, 26]. The reason for male predominance in their peak productive age may be due to the fact that males are more mobile and actively involved in various activities including high risk activities.

Similar to other previous studies [2, 6, 8, 25, 26], majority of injuries were due to RTA. This may be attributable to the legalization of motorcycles as a means of transport in Tanzania, emphasizing the need to have urgent interventions which are specifically aiming at reducing the occurrence of RTA and its related health impacts in this country. Interventional measures at both primary preventive level such as educating motorcyclists and their passengers on adherence to road safety rules and wearing protecting gears as well as secondary prevention such as timely medical and surgical management once individuals sustain injuries should be an enduring focus [7]. Most patients in this study sustained blunt injuries, which is comparable to another previous study in the same city [7], but contrary to another study where penetrating injuries predominated [25].

The anatomical relation of tibia, its subcutaneous orientation with precarious blood supply reiterated on its preponderance involvement in this and other studies [1, 27]. In the present study, nearly two third of patients had Gustilo–Anderson grade II and this is similar to other studies [16, 18] but contrary to a study in Rwanda where Gustilo grade III was the most frequent grade [26]. Moreover, the present study showed a significant association of timing of debridement and Gustilo–Anderson classification with predominance of Gustilo–Anderson grade II.

Despite the prevailing conventional management based on the “6 h rule” especially in developing countries [13], the universally acceptability of this strategy remains elusive. Some studies have shown evidence which advocate the fact that surgical debridement within 6 h may result into favorable outcomes [14, 26, 28–30]; where as others are not supporting timing of debridement as the sore dependent factor predicting favorable outcomes but rather associate the outcomes with the injury severity as per Gustilo–Anderson classification and initial basic interventions [14, 16–18, 20, 31–33]. In the present study, more than half of patients presented late and had surgical debridement done more than six hours after the injury. Late surgical debridement can be explained by late presentation to the hospital due to logistical constraints and poor general condition of the patients that requires pre-operative resuscitation. The overall rate of SSI among the study population (6.3 %) is low and comparable to other studies from USA, Egypt, Canada, UK and Germany where the rates were 14, 11.6, 9.3, 8.5 and 6.6–8 % respectively [15, 18, 27, 30, 34]. The slightly higher infection rates in these countries as opposed to the present study may be related to prolonged follow up period which ranged from 3 to 36 months. Moreover, the rate in the present study is significantly lower than another study in the same hospital where wound sepsis accounted for approximately 22 % connoting improvement in the infection control and prevention for the past three years [35]. The differences may be associated with the environmental conditions where accidents occurred, timing of the interventions as well as surgical techniques in these countries. The renovated accident and emergence department and timely surgical intervention upon arrival of these patients may partly explain the low rate of SSI at BMC in the present study.

In the present study, more cases of SSI were found among patients who had surgical debridement late compare to those who were debrided early (7.5 % versus 4.8 %) although the difference was not statistically significant and is similar to other studies in USA and UK where the respective proportions were 38 % versus 7 % [14], 25 % versus 12 % [30] and 10.8 % versus 10.1 % [36]. This could be attributed to the fact that, delay in prompt management among these patients may create chances of exposure of the fracture site to microbial flora (endogenous) and pathogens (exogenous) leading to SSI. The low infection rates in both groups reiterate the fact that when surgical intervention are promptly done, the outcome may be favorable irrespective of the timing of debridement [15, 18, 31]. Nevertheless, a paradoxical result was found in a study conducted in Egypt where there were of more cases of SSI in the early group (7/12) compared to the late group (5/12) [18]. The length of hospital stay was also slightly more in the late group compared to the early group, although the difference was not statistically significant. In the light of these findings on the outcomes; both groups deserves equal attention so as to reduce financial and other logistical implications related to SSI and longer LOS [32].

In the present study, Gram negative bacteria predominated with *Pseudomonas aeruginosa* being leading bacterial species. Similarly other studies have shown the predominance of Gram negative bacteria [37–39] and of recent *Pseudomonas aeruginosa* is reported at BMC as the commonest bacteria colonizing patients with chronic lower limb ulcers [40]; connoting the possibility of nosocomial transmission if stringent infection control and prevention are not adhered in this setting. Similar to another study done in Uganda, the majority of isolates showed high sensitivities to less used antimicrobial agents (gentamicin, ciprofloxacin, cefepine and meropenem) as opposed to commonly used agents (ampicillin, trimethoprim sulphamethoxazole and ceftriaxone) exemplifying the need to have laboratory guided antimicrobial therapy in this setting [38].

### Study limitation

The follow up of patients was limited to only one months; thus this could have underestimated the incidence of SSI. Anaerobic culture was not done as it is apparently not done at BMC or CUHAS.

## Conclusions

Open long bone fracture injuries are common at BMC, with majority of these injuries being attributed to RTA. The risk of developing SSI was low at BMC and comparable to many other countries; with *Pseudomonas aeruginosa* being commonly implicated causative agent. Despite the fact that there was no statistical significant difference between early versus delayed debrided groups on SSI and LOS stays; the need for prompt surgical intervention in both groups should be an enduring focus to maintain these favorable outcomes.

In the light of this, a long term prospective cohort study with a larger sample size would be of interest to further delineate the predictors of outcome among patients with open long bone fractures as well as the role of bacterial species for effective antimicrobial policy formulation.
